# Long-cap compression hemostasis for puncture-site bleeding after endoscopic injection sclerotherapy: from rescue therapy to routine practice

**DOI:** 10.1055/a-2919-0040

**Published:** 2026-07-29

**Authors:** Nobutaka Doba, Masanobu Someya, Koki Goto, Ryo Yonamine, Shinzo Abe, Shin Maeda

**Affiliations:** 1 Department of Gastroenterology 36998 Yokosuka City HospitalYokosukaKanagawaJapan; 2 Department of GastroenterologyYokohama City University, School of MedicineYokohamaKanagawaJapan

Endoscopic injection sclerotherapy (EIS) remains an effective treatment for esophageal varices because it can obliterate perforating and feeding vessels. 1​ However, puncture-site bleeding is a relatively common adverse event during EIS and may occasionally be difficult to control. 2​ Conventional hemostatic methods for puncture-site bleeding include balloon compression, compression using the needle sheath, and compression using a transparent cap 3​ ​4​ ( Fig. 1 ).

**Fig. 1:**
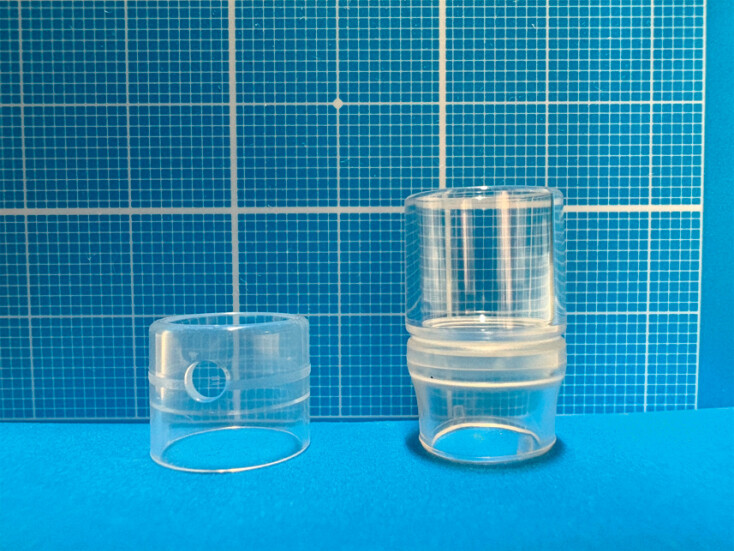
Comparison of transparent caps. **Left** : A conventional transparent cap (protrusion length, 4 mm). **Right** : A long transparent cap (protrusion length, 12 mm) used in the present case.

Nevertheless, the role of cap compression as a rescue technique for puncture-site bleeding refractory to balloon compression has not been sufficiently evaluated. Furthermore, reports describing the routine use of a long cap for post-EIS hemostasis or evaluating the compression time required to achieve hemostasis are scarce.

Here, we present long-cap compression hemostasis for puncture-site bleeding after EIS.

A 54-year-old man with recurrent esophageal varices secondary to alcoholic liver cirrhosis underwent EIS using ethanolamine oleate. Following the first puncture, puncture-site bleeding occurred ( Fig. 2a ). Balloon compression was initially attempted but failed to achieve adequate hemostasis ( Fig. 2b ). Direct compression using a long cap was then performed, resulting in prompt hemostasis 5​ ( Fig. 2c and d ; Video 1 ). During the second puncture in the same session, direct long-cap compression was applied immediately after needle withdrawal, achieving successful hemostasis.

**Fig. 2:**
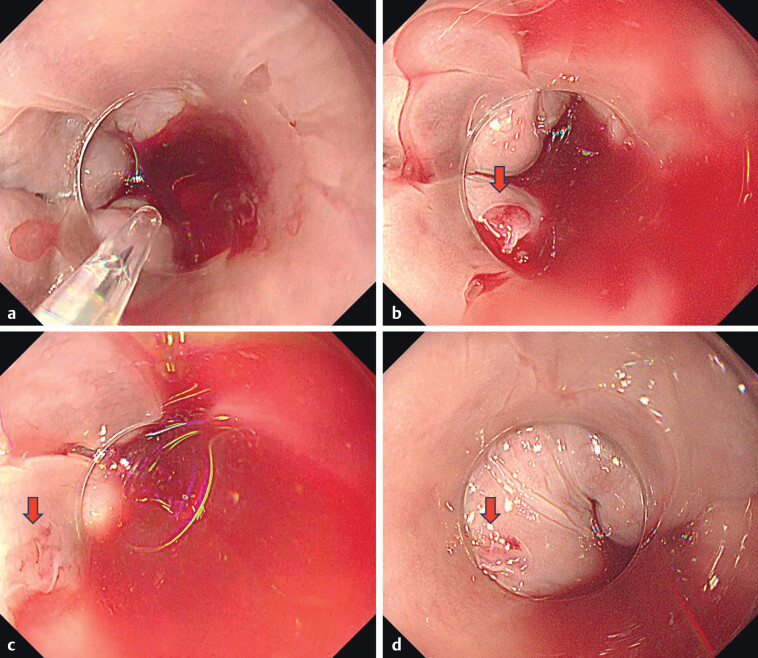
Long-cap compression hemostasis for post-EIS puncture-site bleeding. ( **a** ) Aa active bleeding from the puncture site immediately after needle withdrawal during EIS. ( **b** ) Bleeding persisted despite balloon compression. ( **c** ) Direct compression using a long cap. An observation space was maintained between the endoscope tip and the puncture site, allowing the direct visualization of the bleeding point during compression. ( **d** ) Successful hemostasis following long-cap compression. The arrow indicates the puncture site.

**Video 1** Long-cap compression hemostasis after EIS.


Based on this experience, we adopted long-cap compression as our routine hemostatic method following EIS. In our initial experience involving two patients, three EIS sessions, and nine puncture sites, the mean compression time required to achieve hemostasis was 113.7 ± 56.7 seconds. Hemostasis was achieved at all puncture sites without additional hemostatic interventions, and no delayed bleeding occurred.

The long cap allows the direct visualization of the puncture site during compression, enabling stable hemostasis while confirming cessation of bleeding. Compared with a conventional cap, the long cap provides an adequate observation space during compression, allowing the continuous visualization of the bleeding point. In addition, this technique does not require device exchange or additional fluoroscopic guidance.

This simple and reproducible technique may represent a useful alternative to balloon compression for the management of post-EIS puncture-site bleeding.

Endoscopy_UCTN_Code_CPL_1AH_2AC

## Abbreviation

EIS: Endoscopic injection sclerotherapy

## Patient Consent

The authors confirm that appropriate consent was obtained from the patient for the publication of personal information and images.
